# Supplementary material for: Radiomics strategies for risk assessment of tumour failure in head-and-neck cancer
Source: Sci Rep. 2017 Aug 31;7:10117. doi: 10.1038/s41598-017-10371-5 (PMC5579274; doi:10.1038/s41598-017-10371-5)
Supplement: Supplementary file 1 — Supplementary Info [file 41598_2017_10371_MOESM1_ESM.pdf]

SUPPLEMENTARY INFORMATION

Radiomics strategies for risk assessment of tumour failure in head-and-neck cancer

AUTHORS:
Martin Vallières, Emily Kay-Rivest, Léo Jean Perrin, Xavier Liem, Christophe Furstoss, Hugo J. W. L. Aerts, Nader Khaouam, Phuc Felix Nguyen-Tan, Chang-Shu Wang, Khalil Sultanem, Jan Seuntjens & Issam El Naqa

Preprint submitted to "Scientific Reports" on July 10, 2017

Contents

1 SUPPLEMENTARY RESULTS **4**
1.1 Summary of presentation of results 4
1.2 Choice of complexity of radiomic models 4
1.3 Univariate analysis 4
1.4 Performance of prediction models 7
1.4.1 Complete results: AUC, sensitivity, specificity, accuracy . 7
1.4.2 Complete description of radiomic models 8
1.5 Random forests: radiomic variables only 10
1.6 Final random forest models ans variable importance 11

2 SUPPLEMENTARY METHODS **12**
2.1 Imbalance-adjustment strategy 12
2.2 Construction of radiomic models 13
2.2.1 General workflow 13
2.2.2 Feature set reduction 13
2.2.3 Feature selection 15
2.3 Random forest training 16
2.4 Patient datasets 17
2.4.1 Head and Neck 1 17
2.4.2 Head and Neck 2 20
2.4.3 Head and Neck 3 21
2.4.4 Head and Neck 4 23
2.5 Description of 3D radiomic features 26
2.5.1 First-order statistics (intensity) features 26
2.5.2 Morphological (shape) features 27
2.5.3 Texture features 28
2.5.4 Online resources 36
2.6 Computation of the radiomic signature 37
2.6.1 Original radiomic signature 37
2.6.2 Revised version of the radiomic signature 39

List of Figures

S1 Summary of presentation of results. 5
S2 Choice of complexity of radiomic models. 6
S3 Prediction performance of selected models – complete results. . . 7
S4 Imbalance-adjustment strategy. 12
S5 Workflow of construction of radiomic models. 13
S6 Radiomic feature selection. 15
S7 Random forest training. 16

List of Tables

S1 Univariate analysis of radiomics variables. 6
S2 Univariate analysis of clinical variables. 6
S3 Performance of random forest classifiers constructed using *radiomic* variables only. 10
S4 Best predictive/prognostic and balanced random forest models found in this work. 11
S5 Characteristics of H&N1 cohort – HGJ 17
S6 Characteristics of H&N2 cohort – CHUS 19
S7 Characteristics of H&N3 cohort – HMR 22
S8 Characteristics of H&N4 cohort – CHUM 24

1 SUPPLEMENTARY RESULTS

1.1 Summary of presentation of results

To ease reading and the understanding of this study, a summary of how results are presented in the main text is provided in Supplementary Fig. S1.

1.2 Choice of complexity of radiomic models

From the defined training set of this work (H&N1 and H&N2; $n = 194$) and similarly to the methodology developed in the study of Vallières *et al.*[1], all initial radiomic feature sets (*PET*, *CT* and *PETCT*) first underwent: I) feature set reduction; and II) feature selection of models combining 1 to 10 variables via logistic regression. Prediction performance was then estimated in the training set in terms of the AUC_{632+} metric using the 0.632+ bootstrap resampling technique[2,3], for all the 10 different logistic regression models computed on each of the initial feature sets (Supplementary Fig. S2).

One radiomic model was then chosen for each outcome and feature set, by identifying the lowest number of variables in each model before the prediction performance started reaching a plateau or decreasing (i.e., best parsimonious models). These choices of radiomic model complexity are shown as circles in Supplementary Fig. S2. The logistic regression coefficients forming the final prediction models for these 9 different choices of radiomic models (3 feature sets $\times$ 3 outcomes) were ultimately fitted using the whole training set.

1.3 Univariate analysis

Supplementary Table S1 shows the Spearman's rank correlation coefficients (r_s) between the best PET/CT radiomics variables and the binary outcome vectors for all patients of the four cohorts (H&N1, H&N2, H&N3 and H&N4; $n = 300$). Supplementary Table S2 shows the Spearman's rank correlation coefficients (r_s) between the clinical variables and the binary outcome vectors for all patients of the four cohorts.

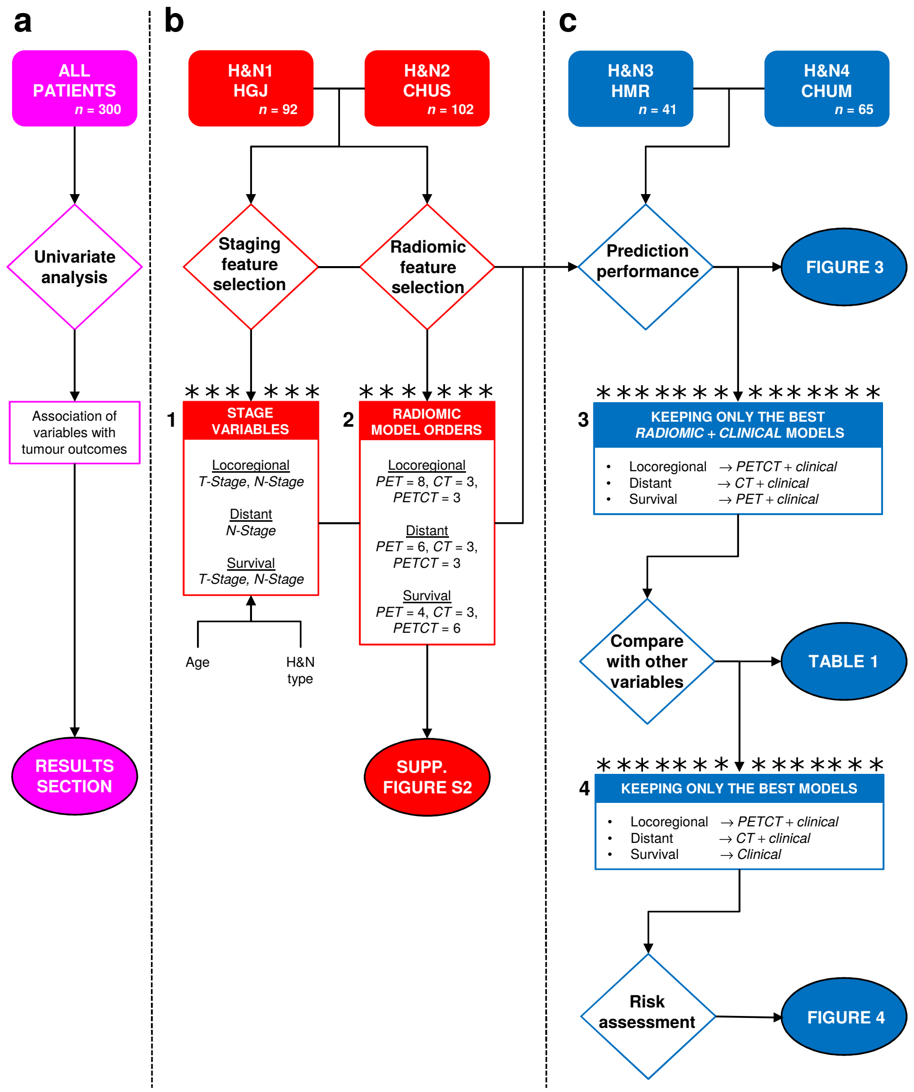

Supplementary Figure S1. Summary of presentation of results. The boxes identified by asterisks represent study checkpoints where only a subset of variables are retained for the remainder of the study. **(a)** Univariate analysis results are computed using the four patient cohorts (H&N1, H&N2, H&N3 and H&N4; $n = 300$) and are presented in the *Results* section of the main text. **(b)** Clinical staging and radiomic feature selection processes are performed using the patient cohorts forming the training set (H&N1 and H&N2; $n = 194$). The clinical staging variables selected for the construction of prediction models are shown in box (1) for each tumour outcome. Radiomic prediction models were selected and built for three initial feature sets: I) PET radiomic features (PET); II) CT radiomic features (CT); and III) PET and CT radiomic features ($PETCT$). Box (2) shows the radiomic models orders (number of combined variables) chosen in Supplementary Fig. S2 for each feature set and outcome. **(c)** Performance of prediction models, comparison with other prognostic factors and risk assessment processes are carried out using the patient cohorts forming the testing set (H&N3 and H&N4; $n = 106$). Prediction performance of all models selected and constructed in the training stage is displayed in Fig. 3 of the main text, and the radiomic feature sets with best prediction performance when combined with clinical variables are shown in box (3) for each outcome. These $radiomic + clinical$ models are further compared against other prognostic factors (e.g. tumour volume, clinical variables alone, etc.) in Table 1. The final three models with best overall prediction/prognostic performance for each outcome are shown in box (4), and only these three models are used to perform outcome risk assessment in Fig. 4 of the main text.

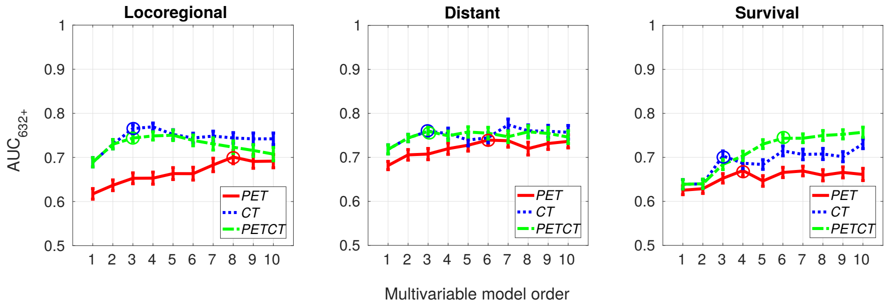

Supplementary Figure S2. Choice of complexity of radiomic models. Choice of the lowest model order (number of combined variables) providing the combination of radiomic variables with the best predictive properties (shown as circles) for each tumour outcome and each of the three initial radiomic feature sets: I) PET radiomic features (PET); II) CT radiomic features (CT); and III) PET and CT radiomic features ($PETCT$). Prediction performance is estimated in the training set (H&N1 and H&N2; $n = 194$) in terms of the AUC_{632+} metric using bootstrap resampling. Error bars represent the standard error of the mean over 100 bootstrap samples.

Supplementary Table S1. Univariate analysis of radiomics variables.

Metric	Locoregional	Distant	Survival
Best PET	(a) $r_s = -0.14, p = 0.02$	(c) $r_s = 0.28, p = 5.8e - 07$*	(e) $r_s = 0.20, p = 3.6e - 04$*
Best CT	(b) $r_s = -0.15, p = 7.3e - 03$	(d) $r_s = -0.29, p = 2.4e - 07$*	(f) $r_s = 0.24, p = 3.7e - 05$*

* Significant associations after multiple testing corrections with a FDR of 10 %.

(a) PET-GLN_{GLSZM}: Scale = 2 mm, Quant. algo = *Uniform*, Ng = 16.

(b) CT-LZHGE_{GLSZM}: Scale = 5 mm, Quant. algo = *Equal*, Ng = 64.

(c) PET-Busyness_{NGTDM}: Scale = 3 mm, Quant. algo = *Uniform*, Ng = 64.

(d) CT-ZSN_{GLSZM}: Scale = 1 mm, Quant. algo = *Uniform*, Ng = 16.

(e) PET-$\text{Coarseness}_{NGTDM}$: Scale = 5 mm, Quant. algo = *Uniform*, Ng = 16.

(f) CT-GLV_{GLRLM}: Scale = 1 mm, Quant. algo = *Uniform*, Ng = 16.

Supplementary Table S2. Univariate analysis of clinical variables.

Metric	Locoregional	Distant	Survival
Age	$r_s = 0.15, p = 7.5e - 03$*	$r_s = -0.03, p = 0.59$	$r_s = -0.14, p = 0.01$*
T-Stage	$r_s = 0.11, p = 0.07$*	$r_s = 0.10, p = 0.09$	$r_s = -0.21, p = 3.0e - 04$*
N-Stage	$r_s = -0.10, p = 0.08$*	$r_s = 0.18, p = 1.4e - 03$*	$r_s = -0.07, p = 0.20$
TNM-Stage	$r_s = -0.09, p = 0.13$	$r_s = 0.09, p = 0.14$	$r_s = -0.08, p = 0.15$
HPV status	$r_s = -0.39, p = 8.0e - 06$*	$r_s = -0.12, p = 0.19$	$r_s = 0.23, p = 0.01$*

* Significant associations after multiple testing corrections with a FDR of 10 %.

1.4 Performance of prediction models

1.4.1 Complete results: AUC, sensitivity, specificity, accuracy

Figure S3a presents the prediction results obtained in the testing set (H&N3 and H&N4; $n = 106$) using the *radiomics* models, and Fig. S3b presents the prediction results obtained in the testing set using the models formed from the combination of radiomic and clinical variables (*radiomics* + *clinical*).

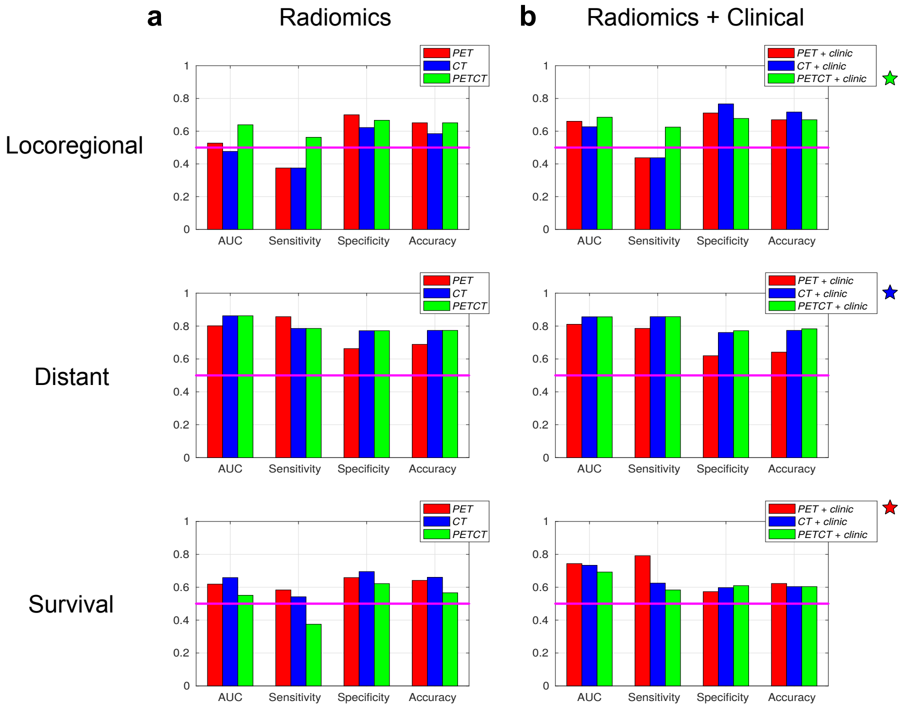

Supplementary Figure S3. Prediction performance of selected models − complete results. All prediction models were selected and built using the training set (H&N1 and H&N2; $n = 194$) for three initial radiomic feature sets: I) PET radiomic features (PET); II) CT radiomic features (CT); and III) PET and CT radiomic features ($PETCT$). The prediction performance is evaluated here for patients of the testing set (H&N3 and H&N4; $n = 106$). **(a)** Prediction performance of radiomic models constructed using logistic regression. **(b)** Prediction performance of radiomic models combined with clinical variables via random forests. The models providing the best overall performance in terms of predictive power and balance of classification of occurence of events and non-occurrence of events are identified with stars.

For locoregional prediction, the model composed of three variables from the $PETCT$ radiomic feature set obtained the best overall performance in terms of predictive power and balance of classification of occurrence of events and non-occurrence of events, with an AUC of 0.64, a sensitivity of 0.56, a specificity of

0.67 and an accuracy of 0.65. The addition of the clinical variables {*Age*, *H&N type*, *T-Stage*, *N-Stage*} to this radiomic model via random forests reached an AUC of 0.69, a sensitivity of 0.63, a specificity of 0.68 and an accuracy of 0.67.

For distant metastases prediction, the best overall performance was obtained with the model composed of three variables from the CT radiomic feature set, with an AUC of 0.86, a sensitivity of 0.79, a specificity of 0.77 and an accuracy of 0.77. The addition of the clinical variables {*Age*, *H&N type*, *N-Stage*} to this radiomic model reached and AUC of 0.86, a sensitivity of 0.86, a specificity of 0.76 and an accuracy of 0.77.

For overal survival prediction (death), the best overall performance was obtained with the model composed of four variables from the PET radiomic feature set, with an AUC of 0.62, a sensitivity of 0.58, a specificity of 0.66 and an accuracy of 0.64. The addition of the clinical variables {*Age*, *H&N type*, *T-Stage*, *N-Stage*} to this radiomic model reached and AUC of 0.74, a sensitivity of 0.79, a specificity of 0.57 and an accuracy of 0.62.

1.4.2 Complete description of radiomic models

This section provides the complete description (specific features, texture extraction parameters, logistic regression coefficients) of the three best radiomics models of this work, one for each outcome. Significance of the variables in the logistic regression models constructed from the training set (H&N1 and H&N2; $n = 194$) was assessed using the Wald's test implemented in the software DREES[4].

→ Locoregional recurrence

- PET-GLN$_{GLSZM}$: Scale = 2 mm, Quant. algo = *Uniform*, Ng = 64
- CT-Correlation$_{GLCM}$: Scale = 1 mm, Quant. algo = *Uniform*, Ng = 16
- CT-LGZE$_{GLSZM}$: Scale = 1 mm, Quant. algo = *Equal*, Ng = 8
- Significance of variables: $p = 0.04$, $p = 0.004$, $p = 0.02$
- Complete multivariable model response:

$$g(\mathbf{x}_i) = -350.1 \times \text{PET-GLN}_{GLSZM} + 7.42 \times \text{CT-Correlation}_{GLCM} + 21.14 \times \text{CT-LGZE}_{GLSZM} - 0.635$$

→ Distant metastases

- CT-LRHGE$_{GLRLM}$: Scale = 1 mm, Quant. algo = *Equal*, Ng = 8
- CT-ZSV$_{GLSZM}$: Scale = 5 mm, Quant. algo = *Equal*, Ng = 8
- CT-ZSN$_{GLSZM}$: Scale = 1 mm, Quant. algo = *Uniform*, Ng = 16

- Significance of variables: $p = 0.03$, $p = 0.03$, $p = 0.03$
- Complete multivariable model response:

$$g(\mathbf{x}_i) = 0.0233 \times \text{CT-LRHGE}_{GLRLM} - 226.7 \times \text{CT-ZSV}_{GLSZM} - 14.9 \times \text{CT-ZSN}_{GLSZM} + 1.21$$

→ **Overall survival (death)**

- PET-LGRE$_{GLRLM}$: Scale = 4 mm, Quant. algo = *Equal*, Ng = 64
- PET-SZE$_{GLSZM}$: Scale = 3 mm, Quant. algo = *Uniform*, Ng = 16
- PET-HGZE$_{GLSZM}$: Scale = 1 mm, Quant. algo = *Uniform*, Ng = 64
- PET-ZSN$_{GLSZM}$: Scale = 1 mm, Quant. algo = *Equal*, Ng = 8
- Significance of variables: $p = 0.2$, $p = 0.009$, $p = 0.04$, $p = 0.2$
- Complete multivariable model response:

$$g(\mathbf{x}_i) = -136.8 \times \text{PET-LGRE}_{GLRLM} + 11.49 \times \text{PET-SZE}_{GLSZM} - 0.0035 \times \text{PET-HGZE}_{GLSZM} - 25.91 \times \text{PET-ZSN}_{GLSZM} + 3.921$$

1.5 Random forests: radiomic variables only

Supplementary Table S3. Performance of random forest classifiers constructed using *radiomic* variables only.

Outcome	Selected features[a]	Prediction				Prognosis	
		AUC[b]	Sensitivity[b]	Specificity[b]	Accuracy[b]	CI[c]	p-value[d]
Locoregional	PET-GLN$_{GLSZM}$<br>CT-Correlation$_{GLCM}$<br>CT-LGZE$_{GLSZM}$	0.61	0.56	0.68	0.66	0.60	0.16
Distant	CT-LRHGE$_{GLRLM}$<br>CT-ZSV$_{GLSZM}$<br>CT-ZSN$_{GLSZM}$	0.86	0.79	0.77	0.77	0.88	0.000007
Survival	PET-LGRE$_{GLRLM}$<br>PET-SZE$_{GLSZM}$<br>PET-HGZE$_{GLSZM}$<br>PET-ZSN$_{GLSZM}$	0.60	0.71	0.45	0.51	0.58	0.28

[a] See Supplementary Results section 1.4.2 for the list of extraction parameters of texture features.

[b] Binary prediction of outcome using random forest probability output.

[c] Concordance-index between random forest probability output and time to event.

[d] Log-rank test from Kaplan-Meier curves with a risk stratification into two groups (probability threshold of 0.5).

1.6 Final random forest models ans variable importance

In Supplementary Table S4, the features of the three final random forest models developed in this work are listed by order of importance in the models. To assess the importance of each feature in each model, an approach combining random permutations and bootstrap resampling was used. First, 100 bootstrap samples were drawn from the testing set (H&N3 and H&N4 cohorts; $n = 106$). For each bootstrap sample, the feature values of all patients of the testing set were permuted once (same permutation for all features). The average percent AUC change over all permutations was then calculated by comparing random permutation AUCs of each variable separately to the true bootstrap AUCs. Significance of each variable in the model (p-value) was calculated by comparing the distribution of true bootstrap AUCs to the distribution of permuted AUCs via the Wilcoxon right-sided test. The more the AUC decreases as a result of random permutations, the more important the variable is to the model.

Supplementary Table S4. Best predictive/prognostic and balanced random forest models found in this work.

Outcome	Selected features[a]	AUC change[b]	p-value[c]
Locoregional	Age	−22.8%	$\ll 0.001$
	CT-LGZE$_{GLSZM}$	−16.3%	$\ll 0.001$
	PET-GLN$_{GLSZM}$	−16.1%	$\ll 0.001$
	CT-Correlation$_{GLCM}$	−14.6%	$\ll 0.001$
	H&N type	−14.2%	$\ll 0.001$
	N-Stage	−13.4%	$\ll 0.001$
	T-Stage	−12.3%	$\ll 0.001$
Distant	CT-ZSN$_{GLSZM}$	−15.9%	$\ll 0.001$
	CT-ZSV$_{GLSZM}$	−7.7%	$\ll 0.001$
	CT-LRHGE$_{GLRLM}$	−3.1%	0.00002
	H&N type	+0.2%	0.40
	N-Stage	+2.7%	1
	Age	+3.5%	1
Survival	H&N type	−13.8%	$\ll 0.001$
	T-Stage	−9.9%	$\ll 0.001$
	Age	−9.6%	$\ll 0.001$
	N-Stage	−1.2%	0.30

[a] See Supplementary Results section 1.4.2 for the list of extraction parameters of texture features.

[b] Average of $(AUC_{perm} - AUC_{true})/AUC_{true}$ over all permutations. The more negative, the more important the variable is in the model.

[c] Significance in the model via the Wilcoxon right-sided test.

2 SUPPLEMENTARY METHODS

2.1 Imbalance-adjustment strategy

In Supplementary Fig. S4, the imbalance-adjustment strategy used in this work is detailed. In our work, this strategy is combined to uniform bootstrap resampling: every time a boostrap sample is created for prediction estimation using logistic regression or for random forest construction, an ensemble of multiple balanced classifiers is used (in contrast to using only one unbalanced classifier).

In Supplementary Fig. S4, please note that "$[x]$" refers to a rounding operation, "$\lceil x \rceil$" refers to a ceiling operation, and "$\lfloor x \rfloor$" refers to a floor operation. For example, for $N- = 56$ and $N+ = 11$, 5 partitions would be created. All partitions would contain the initial 11 positive instances. The 56 negative instances would be distributed between the 5 partitions such that the first 4 partitions would contain 11 negative instances and the last one 12 negative instances.

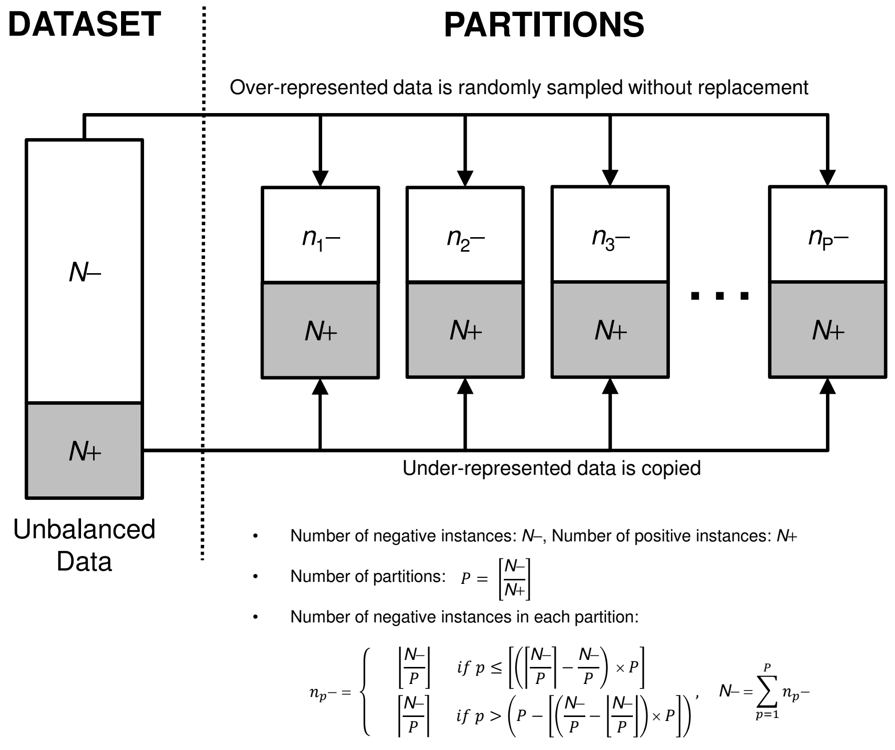

Supplementary Figure S4. Imbalance-adjustment strategy. Adapted from Schiller *et al.*[5]

2.2 Construction of radiomic models

2.2.1 General workflow

Supplementary Fig. S5 presents the general wokflow used to construct radiomic models. For more details, please see the next two sections and the work of Vallières *et al.*[1]

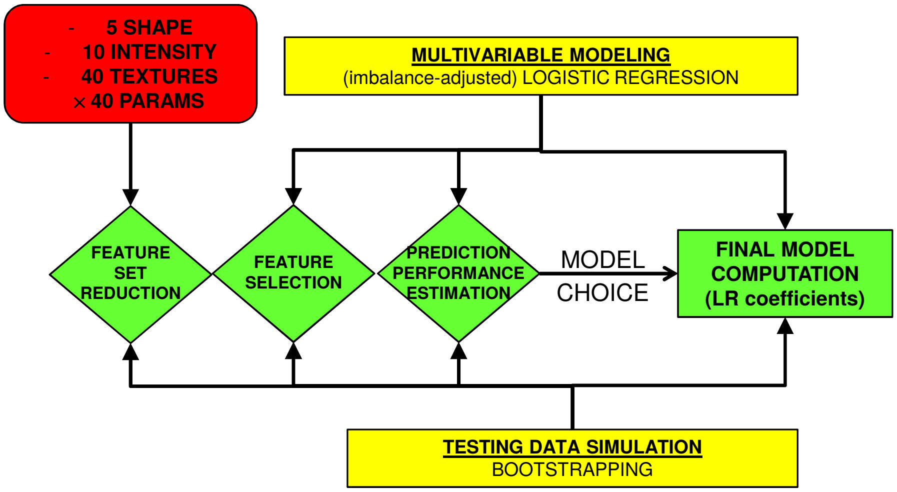

Supplementary Figure S5. Workflow of construction of radiomic models.

2.2.2 Feature set reduction

Feature set reduction is performed for each of the initial feature sets via a stepwise forward feature selection scheme to create reduced feature sets containing 25 different scan-texture features (or non-texture features) balanced between predictive power and non-redundancy. This procedure is carried out using the following *Gain* equation:

$$
\begin{aligned}
\widehat{\text{Gain}}_j &= \gamma \cdot |\widehat{r}_s(\mathbf{x}_j, \mathbf{y})| \\
&\quad + \delta_a \cdot \left[\sum_{k=1}^{f} \left(\frac{2(f-k+1)}{f(f+1)} \right) \widehat{\text{PIC}}(\mathbf{x}_k, \mathbf{x}_j) \right] \\
&\quad + \delta_b \cdot \left[\frac{1}{F} \sum_{l=1}^{F} \widehat{\text{PIC}}(\mathbf{x}_l, \mathbf{x}_j) \right], \qquad (1) \\
&\text{where} \quad \widehat{r}_s(\mathbf{x}_j, \mathbf{y}) = \frac{1}{B} \sum_{b=1}^{B} r_s(\mathbf{x}_j^{*b}, \mathbf{y}), \\
&\text{and} \quad \widehat{\text{PIC}}(\mathbf{x}_k, \mathbf{x}_j) = \frac{1}{B} \sum_{b=1}^{B} \text{PIC}(\mathbf{x}_k^{*b}, \mathbf{x}_j^{*b}).
\end{aligned}
$$

In equation (1), $r_s(\mathbf{x}_j, \mathbf{y})$ is the Spearman's rank correlation computed between a given feature vector $\mathbf{x}_j$ and an outcome vector $\mathbf{y}$. $\text{PIC}(\mathbf{x}_k, \mathbf{x}_j)$ is the *potential information coefficient* defined as $\text{PIC}(\mathbf{x}_k, \mathbf{x}_j) = 1 - \text{MIC}(\mathbf{x}_k, \mathbf{x}_j)$, where $\text{MIC}(\mathbf{x}_k, \mathbf{x}_j)$ is the *maximal information coefficient*[6] between feature k and j. The sum over k is a sum over all f features that have already been chosen to be part of the reduced feature set (employed in forward selection schemes), whereas the sum over l is a sum over all F features that have not yet been removed from a larger initial set (employed in backward selection schemes). The sum over the k features is always done in order of appearance of the different features in the reduced set in order to favour the features from the larger initial set with the least dependence with the features chosen first in the reduced set. In this work, γ was set to 0.5, δ_a to 0.5 and δ_b to 0. Every time a new feature was chosen in the reduced set from a larger initial set, a new *Gain* was calculated for all remaining features in the larger initial set using a different set of 100 bootstrap samples ($*b$, with $b = 1, \ldots, B$). Note that equation (1) allows to rank specific scan-texture-parameter features, as part 1 of the *Gain* equation uses Spearmans rank correlations varying over the whole set of texture extraction parameters. However, to speed up calculations, average scan-texture features over all texture extraction parameters were used in part 2 (and 3 if needed) of the *Gain* equation.

2.2.3 Feature selection

The feature selection step is first divided into 25 experiments. In each of these experiments, a different feature from a given reduced set is used as a different "starting feature". For a given starting feature, all possible logistic regression models of order 2 (i.e. combination of 2 variables) are created by combining that feature with each of the remaining features in the reduced feature set still available for that particular experiment. Bootstrap resampling (100 samples) is performed for each of these models in order to calculate the 0.632+ bootstrap AUC[2,3], a process in which logistic regression models are trained in bootstrap training samples and tested in corresponding bootstrap testing samples. Then, the single remaining feature that maximizes the 0.632+ bootstrap AUC when combined with the starting feature is selected, and the process is repeated up to model order 10 for each experiment. Finally, for each model order, the experiment that yields the highest 0.632+ bootstrap AUC is identified, and combinations of features are thereby selected for model orders of 1 to 10.

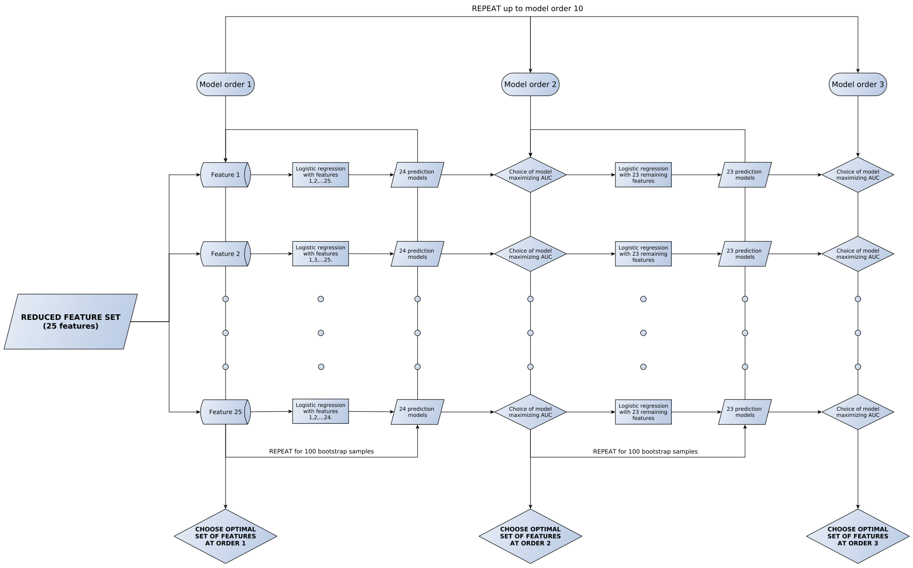

Supplementary Figure S6. Radiomic feature selection.

2.3 Random forest training

Supplementary Fig. S7 presents the methodology used in this work for random forest training. Stratified random sub-sampling is used to estimate the predictive properties of the random forests (e.g. estimating the best tumour staging metric addition and positive instances weight in the forests by maximizing $\widehat{\text{AUC}}$). For each training sub-sample, boostrap resampling is used to grow a single random forest to be tested in the corresponding testing sub-sample. Through the imbalance-adjustment strategy, each bootstrap sample produces multiple decision trees (one decision tree per partition) to be appended to the random forest of the corresponding training sub-sample (in contrast to conventionally producing a single decision tree per bootstrap sample).

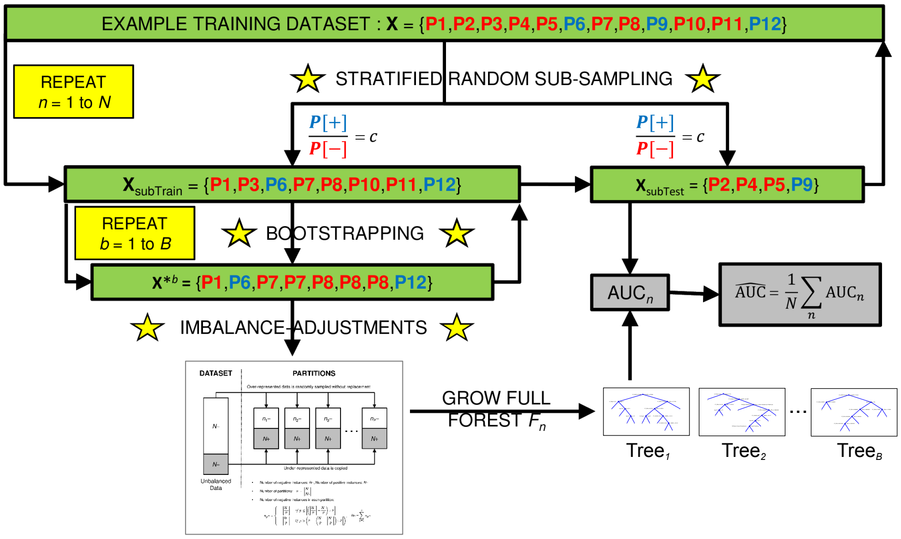

Supplementary Figure S7. Random forest training.

2.4 Patient datasets

2.4.1 Head and Neck 1

→ Hôpital général juif, Montréal, QC

Supplementary Table S5. Characteristics of H&N1 cohort – HGJ

Characteristic	Type	No. of patients
Gender	Male	75 (82 %)
	Female	17 (18 %)
Age	Range	18-84
	Mean ± STD	61 ± 11
Tumour type	Oropharynx	56 (61 %)
	Hypopharynx	4 (4 %)
	Nasopharynx	14 (15 %)
	Larynx	14 (15 %)
	Unknown	4 (4 %)
T-Stage	T1	20 (22 %)
	T2	20 (22 %)
	T3	35 (38 %)
	T4	13 (14 %)
	Tx	4 (4 %)
N-Stage	N0	13 (14 %)
	N1	18 (20 %)
	N2	58 (63 %)
	N3	3 (3 %)
TNM-Stage	Stage I	1 (1 %)
	Stage II	5 (5 %)
	Stage III	28 (30 %)
	Stage IV	58 (63 %)
HPV status	Positive	30 (33 %)
	Negative	25 (27 %)
	N/A	37 (40 %)
Treatment	Radiation only	5 (5 %)
	Chemo-radiation	87 (95 %)
Outcome	Locoregional recurrence	12 (13 %)
	Distant metastases	16 (17 %)
	Death	14 (15 %)

PATIENT POPULATION. This cohort is composed of 92 patients with primary squamous cell carcinoma of the head-and-neck (stage I-IVb) treated between 2006 and 2014 at Hôpital général juif, Montréal, QC. Included patients were treated with curative intent with radiation alone or with chemo-radiation. Patients with recurrent head-and-neck cancer or with metastases at presentation, and patients receiving palliative treatment were excluded from the study. The median follow-up period of the cohort was 46 months (range: 11-112). Pa-

tients that did not develop a locoregional recurrence or distant metastases during the follow-up period and that had a follow-up time smaller than 24 months were also excluded from the study. The study has been approved by the institutional review board of Hôpital général juif. Detailed information about this patient cohort is provided in Supplementary Table S5.

TREATMENT DETAILS. Patients with stage I-II disease were treated with definitive radiotherapy alone while patients with stage III-IV disease were treated using concurrent chemo-radiation. The radiotherapy regimen was planned using Volumetric Arc Modulated Radiotherapy Rapidarc planning system (Varian Medical Systems). The radiotherapy regime consisted of hypofractionated fractionated radiotherapy with simultaneous integrated boost where the GTV was planned to receive a total of 67.5 Gy in fractions of 2.25 Gy over 6 weeks, while CTV received a total of 54-60 Gy in fractions of 1.8-2 Gy over 30 fractions. The treatment was delivered on a Linac equipped with HD120 Multileaf Collimator, with Image Guided Radiotherapy using daily kv-kv imaging and weekly Cone beam CT-scan (CBCT). Concomitant chemotherapy was given via weekly administration of Carboplatin at AUC 2-3 and Paclitaxel at dose of 40 mg/m^2.

FDG-PET/CT SCANS. All 92 eligible patients had FDG-PET and CT scans done on a hybrid PET/CT scanner (Discovery ST, GE Healthcare) within 37 days before treatment (median: 14 days). For the PET portion of the FDG-PET/CT scan, a median of 584 MBq (range: 368-715) was injected intravenously. Imaging acquisition of the head and neck was performed using multiple bed positions with a median of 300 s (range: 180-420) per bed position. Attenuation corrected images were reconstructed using an ordered subset expectation maximization (OSEM) iterative algorithm and a span (axial mash) of 5. The FDG-PET slice thickness resolution was 3.27 mm for all patients and the median in-plane resolution was 3.52×3.52 mm^2 (range: 3.52-4.69). For the CT portion of the FDG-PET/CT scan, an energy of 140 kVp with an exposure of 12 mAs was used. The CT slice thickness resolution was 3.75 mm and the median in-plane resolution was 0.98×0.98 mm^2 for all patients. Contours defining the gross tumour volume (GTV) and lymph nodes were drawn by an expert radiation oncologist in a radiotherapy treatment planning system. For 2 of the 92 patients, the radiotherapy contours were directly drawn on the CT scan of the FDG-PET/CT scan. For 90 of the 92 patients, the radiotherapy contours were drawn on a different CT scan dedicated to treatment planning. In the latter case, the contours were propagated to the FDG-PET/CT scan reference frame using deformable registration with the software MIM® (MIM software Inc., Cleveland, OH).

Supplementary Table S6. Characteristics of H&N2 cohort – CHUS

Characteristic	Type	No. of patients
Gender	Male	74 (73 %)
	Female	28 (27 %)
Age	Range	34-88
	Mean ± STD	64 ± 10
Tumour type	Oropharynx	73 (72 %)
	Hypopharynx	1 (1 %)
	Nasopharynx	6 (6 %)
	Larynx	22 (22 %)
T-Stage	T1	9 (9 %)
	T2	45 (44 %)
	T3	31 (30 %)
	T4	17 (17 %)
N-Stage	N0	38 (37 %)
	N1	11 (11 %)
	N2	50 (49 %)
	N3	3 (3 %)
TNM-Stage	Stage I	3 (3 %)
	Stage II	17 (17 %)
	Stage III	22 (22 %)
	Stage IV	60 (59 %)
HPV status	Positive	26 (25 %)
	Negative	13 (13 %)
	N/A	63 (62 %)
Treatment	Radiation only	33 (32 %)
	Chemo-radiation	69 (68 %)
Outcome	Locoregional recurrence	17 (17 %)
	Distant metastases	10 (10 %)
	Death	18 (18 %)

2.4.2 Head and Neck 2

→ Centre hospitalier universitaire de Sherbooke, Sherbrooke, QC

PATIENT POPULATION. This cohort is composed of 102 patients with primary squamous cell carcinoma of the head-and-neck (stage I-IVb) treated between 2007 and 2014 at Centre hospitalier universitaire de Sherbooke, Sherbrooke, QC. Included patients were treated with curative intent with radiation alone or with chemo-radiation. Patients with recurrent head-and-neck cancer or with metastases at presentation, and patients receiving palliative treatment were excluded from the study. The median follow-up period of the cohort was 44 months (range: 8-93). Patients that did not develop a locoregional recurrence or distant metastases during the follow-up period and that had a follow-up time smaller than 24 months were also excluded from the study. The study has been approved by the institutional review board of Centre hospitalier universitaire de Sherbooke. Detailed information about this patient cohort is provided in Supplementary Table S6.

TREATMENT DETAILS. All patients have had a pathological confirmation of squamous cell carcinoma and imaging examination for tumor staging before all treatments. All those patients have had a treatment position PET imaging in our center. The PET images have been merged with dosimetry CT imaging, and the dosimetry plan has been performed with teraplan for 3D-conformal technique and pinnacle system for IMRT. The 3D-conformal technique has been used for all patients before 2008, and since 2008, all patients have been treated by IMRT. The treatment approaches consisted of either radiotherapy alone or radiotherapy with concurrent chemotherapy or concurrent Cetuximab. The treatment dose varied according to the tumor staging. The patients with T1 glottic laryngeal cancer have been treated mostly by 2.5 Gy daily for total dose of 50Gy, some patients have been treated with daily dose of 2.25 Gy for 63 Gy totally. All other patients with T1, T2, N0 cancers have been treated with standard fractionated radiation schedules of 60-66 Gy; for the patients with T3-4, or N+, the treatment dose varied from 68.8 Gy in 32 fractions to 70 Gy in 33 fractions. All treatments have been performed by 6 MV linear accelerator. The concurrent chemotherapy was either cisplatin 100 mg/m^2 at D1, D22 & D43, or cisplatin 40 mg/m^2, weekly. According to the consideration of the oncologist, some patients have been treated by radiotherapy associated with Cetuximab, due to the problems of kidney function, audition, elder or weak general performance status. The treatment schedule of concurrent Cetuximab was administrated according to the study of Bonner *el al.*[7]

FDG-PET/CT SCANS. All 102 eligible patients had FDG-PET and CT scans done on a hybrid PET/CT scanner (GeminiGXL 16, Philips) within 54 days before treatment (median: 19 days). For the PET portion of the FDG-PET/CT scan, a median of 325 MBq (range: 165-517) was injected intravenously. Imaging acquisition of the head and neck was performed using multiple bed posi-

tions with a median of 150 s (range: 120-151) per bed position. Attenuation corrected images were reconstructed using a LOR-RAMLA iterative algorithm. The FDG-PET slice thickness resolution was 4 mm and the median in-plane resolution was 4×4 mm^2 for all patients. For the CT portion of the FDG-PET/CT scan, a median energy of 140 kVp (range: 12-140) with a median exposure of 210 mAs (range: 43-250) was used. The median CT slice thickness resolution was 3 mm (range: 2-5) and the median in-plane resolution was 1.17×1.17 mm^2 (range: 0.68-1.17). Contours defining the gross tumour volume (GTV) and lymph nodes were drawn by an expert radiation oncologist in a radiotherapy treatment planning system. For 91 of the 102 patients, the radiotherapy contours were directly drawn on the CT scan of the FDG-PET/CT scan. For 11 of the 102 patients, the radiotherapy contours were drawn on a different CT scan dedicated to treatment planning. In the latter case, the contours were propagated to the FDG-PET/CT scan reference frame using deformable registration with the software MIM® (MIM software Inc., Cleveland, OH).

2.4.3 Head and Neck 3

→ Hôpital Maisonneuve-Rosemont, Montréal, QC

PATIENT POPULATION. This cohort is composed of 41 patients with primary squamous cell carcinoma of the head-and-neck (stage II-IVb) treated between 2008 and 2014 at Hôpital Maisonneuve-Rosemont, Montréal, QC. Included patients were treated with curative intent with radiation alone or with chemo-radiation. Patients with recurrent head-and-neck cancer or with metastases at presentation, and patients receiving palliative treatment were excluded from the study. The median follow-up period of the cohort was 38 months (range: 6-70). Patients that did not develop a locoregional recurrence or distant metastases during the follow-up period and that had a follow-up time smaller than 24 months were also excluded from the study. The study has been approved by the institutional review board of Hôpital Maisonneuve-Rosemont. Detailed information about this patient cohort is provided in Supplementary Table S7.

TREATMENT DETAILS. The treatment options consisted of either definitive radiotherapy alone or concurrent chemo-radiation. All patients received continuous course of radiotherapy delivered by a 6 MV linear accelerator using 7 to 9 fields inverse planning IMRT. Only one patient was planned with 5 fields and another was treated using 6 fields forward planning IMRT to the upper neck and direct anterior field with a spinal cord block to the lower neck. For the patients receiving radiotherapy alone, 4 patients had stage II disease including a T1N1 nasopharyngeal cancer and received a dose 69.96 Gy in 33 fractions, 2 oropharyngeal and 1 hypopharyngeal cancer receiving altered fractionation with a dose of 66 to 67.5 Gy in 30 fractions. The 3 patients were offered but declined the chemotherapy and received 69.36 Gy in 33 fractions. Among patients receiving chemo-radiation, the radiation fractionation mostly used was 69.96 Gy in 33 fractions ($n = 31$) and the remaining received 70 Gy in 35 fractions ($n = 2$).

Supplementary Table S7. Characteristics of H&N3 cohort – HMR

Characteristic	Type	No. of patients
Gender	Male	31 (76 %)
	Female	10 (24 %)
Age	Range	49-85
	Mean ± STD	67 ± 9
Tumour type	Oropharynx	19 (46 %)
	Hypopharynx	7 (17 %)
	Nasopharynx	6 (15 %)
	Larynx	9 (22 %)
T-Stage	T1	2 (5 %)
	T2	17 (41 %)
	T3	9 (22 %)
	T4	12 (29 %)
	Tx	1 (2 %)
N-Stage	N0	5 (12 %)
	N1	4 (10 %)
	N2	27 (66 %)
	N3	5 (12 %)
TNM-Stage	Stage I	0 (0 %)
	Stage II	3 (7 %)
	Stage III	5 (12 %)
	Stage IV	33 (80 %)
HPV status	Positive	2 (5 %)
	Negative	0 (0 %)
	N/A	39 (95 %)
Treatment	Radiation only	7 (17 %)
	Chemo-radiation	34 (83 %)
Outcome	Locoregional recurrence	9 (22 %)
	Distant metastases	11 (27 %)
	Death	19 (46 %)

The concurrent chemotherapy was in most cases cisplatin 100 mg/m2 i.v. every 3 weeks.

FDG-PET/CT SCANS. All 41 eligible patients had FDG-PET and CT scans done on a hybrid PET/CT scanner (Discovery STE, GE Healthcare) within 60 days before treatment (median: 34 days). For the PET portion of the FDG-PET/CT scan, a median of 475 MBq (range: 227-859) was injected intravenously. Imaging acquisition of the head and neck was performed using multiple bed positions with a median of 360 s (range: 120-360) per bed position. Attenuation corrected images were reconstructed using an ordered subset expectation maximization (OSEM) iterative algorithm and a median span (axial mash) of 5 (range: 3-5). The FDG-PET slice thickness resolution was 3.27 mm for all patients and the median in-plane resolution was 3.52×3.52 mm^2 (range: 3.52-5.47). For the CT portion of the FDG-PET/CT scan, a median energy of 140 kVp (range: 120-140) with a median exposure of 11 mAs (range: 5-16) was used. The CT slice thickness resolution was 3.75 mm for all patients and the median in-plane resolution was 0.98×0.98 mm^2 (range: 0.98-1.37). For all 41 patients, the radiotherapy contours defining the gross tumour volume (GTV) and lymph nodes were drawn by an expert radiation oncologist on a different CT scan dedicated to treatment planning. The contours were then propagated to the FDG-PET/CT scan reference frame using deformable registration with the software MIM® (MIM software Inc., Cleveland, OH).

2.4.4 Head and Neck 4

→ Centre hospitalier de l'Université de Montréal, Montréal, QC

PATIENT POPULATION. This cohort is composed of 65 patients with primary squamous cell carcinoma of the head-and-neck (stage II-IVb) treated between 2009 and 2013 at Centre hospitalier de l'Université de Montréal, Montréal, QC. Included patients were treated with curative intent with radiation alone or with chemo-radiation. Patients with recurrent head-and-neck cancer or with metastases at presentation, and patients receiving palliative treatment were excluded from the study. The median follow-up period of the cohort was 40 months (range: 11-66). Patients that did not develop a locoregional recurrence or distant metastases during the follow-up period and that had a follow-up time smaller than 24 months were also excluded from the study. The study has been approved by the institutional review board of Centre hospitalier de l'Université de Montréal. Detailed information about this patient cohort is provided in Supplementary Table S8.

TREATMENT DETAILS. Most patients (94 %) underwent concurrent platinum based chemotherapy and radiotherapy. All patients received an IMRT type radiation (sliding window IMRT or tomotherapy) consisting of 70 Gy of radiation in 33 fractions. Immobilisation device included a thermoplastic mask of the head and shoulder fixed to the treatment table.

Supplementary Table S8. Characteristics of H&N4 cohort – CHUM

Characteristic	Type	No. of patients
Gender	Male	49 (75 %)
	Female	16 (25 %)
Age	Range	44-90
	Mean ± STD	63 ± 9
Tumour type	Oropharynx	58 (89 %)
	Hypopharynx	0 (0 %)
	Nasopharynx	2 (3 %)
	Larynx	0 (0 %)
	Unknown	5 (8 %)
T-Stage	T1	8 (12 %)
	T2	28 (43 %)
	T3	19 (29 %)
	T4	5 (8 %)
	Tx	5 (8 %)
N-Stage	N0	4 (6 %)
	N1	8 (12 %)
	N2	45 (69 %)
	N3	8 (12 %)
TNM-Stage	Stage I	0 (0 %)
	Stage II	2 (3 %)
	Stage III	7 (11 %)
	Stage IV	54 (83 %)
	N/A	2 (3 %)
HPV status	Positive	21 (32 %)
	Negative	3 (5 %)
	N/A	41 (63 %)
Treatment	Radiation only	4 (6 %)
	Chemo-radiation	61 (94 %)
Outcome	Locoregional recurrence	7 (11 %)
	Distant metastases	3 (5 %)
	Death	5 (8 %)

FDG-PET/CT SCANS. All 65 eligible patients had FDG-PET and CT scans done on a hybrid PET/CT scanner (Discovery STE, GE Healthcare) within 66 days before treatment (median: 12 days). For the PET portion of the FDG-PET/CT scan, a median of 315 MBq (range: 199-3182) was injected intravenously. Imaging acquisition of the head and neck was performed using multiple bed positions with a median of 300 s (range: 120-420) per bed position. Attenuation corrected images were reconstructed using an ordered subset expectation maximization (OSEM) iterative algorithm and a medianspan (axial mash) of 3 (range: 3-5). The median FDG-PET slice thickness resolution was 4 mm (range: 3.27-4) and the median in-plane resolution was 4×4 mm^2 (range: 3.52-5.47). For the CT portion of the FDG-PET/CT scan, a median energy of 120 kVp (range: 120-140) with a median exposure of 350 mAs (range: 5-350) was used. The median CT slice thickness resolution was 1.5 mm (range: 1.5-3.75) and the median in-plane resolution was 0.98×0.98 mm^2 (range: 0.98-1.37). All patients received their FDG-PET/CT scan dedicated to the head and neck area right before their planning CT scan, in the same position with the immobilisation device. Contours defining the gross tumour volume (GTV) and lymph nodes were drawn by an expert radiation oncologist on the planning CT scan. The contours were then propagated to the FDG-PET/CT scan reference frame using deformable registration with the software MIM® (MIM software Inc., Cleveland, OH) to ensure proper coverage.

2.5 Description of 3D radiomic features

In this work, a total of 10 first-order statistics (intensity) features, 5 morphological (shape) features and 40 texture features extracted using 40 different parameters were computed for both the separate PET and CT volumes of diagnostic FDG-PET/CT scans (unless otherwise noted). The definition of the region of interest (ROI) on both PET and CT volumes was the original sub-volume "$\text{GTV}_{\text{primary}} + \text{GTV}_{\text{lymph nodes}}$" as drawn on CT planning scans for radiotherapy purposes and deformably registered to the diagnostic FDG-PET/CT scans.

It is recommended to extract all radiomic features from an input volume with isotropic voxel size (prior resampling may be required) in order for the number of voxel measurements and distance parameters used for feature extraction to be meaningful in 3D space and for the orientation dependence of the tumour to be minimized.

2.5.1 First-order statistics (intensity) features

Let P define the first-order histogram of a volume $V(x, y, z)$ with isotropic voxel size. $P(i)$ represents the number of voxels with gray-level i, and N_g represents the number of gray-level bins set for P. The ith entry of the normalized histogram is then defined as:

$$p(i) = \frac{P(i)}{\sum_{i=1}^{N_g} P(i)}.$$

The first-order statistics features (10) are then defined as:

- **Variance**:

$$\sigma^2 = \sum_{i=1}^{N_g} (i - \mu)^2 \, p(i)$$

- **Skewness**:

$$s = \sigma^{-3} \sum_{i=1}^{N_g} (i - \mu)^3 \, p(i)$$

- **Kurtosis**:

$$k = \sigma^{-4} \sum_{i=1}^{N_g} \left[(i - \mu)^4 \, p(i) \right] - 3$$

- **SUVmax**: Maximum SUV of the tumour region. Extracted from PET scans and not used in the CT feature set.
- **SUVpeak**: Average of the voxel with maximum SUV within the tumour region and its 26 connected neighbours. Extracted from PET scans and not used in the CT feature set.

- **SUVmean**: Average SUV value of the tumour region. Extracted from PET scans and not used in the *CT* feature set.

- **AUC-CSH**: Area under the curve of the cumulative SUV-volume histogram describing the percentage of total tumour volume above a percentage threshold of maximum SUV, as defined by van Velden *et al.*[8] Extracted from PET scans and not used in the *CT* feature set.

- **TLG**: Total lesion glycolysis. Defined as SUVmean × total volume of the tumour region. Extracted from PET scans and not used in the *CT* feature set.

- **Percent Inactive**: Percentage of the tumour region that is inactive. As defined by Vallières *et al.*[1], a threshold of $0.01 \times (\text{SUVmax})^2$ followed by closing and opening morphological operations were used to differentiate active and inactive regions on FDG-PET scans. Extracted from PET scans and not used in the *CT* feature set.

- **gETU**: Generalized effective total uptake, with parameter $a = 0.25$ as defined by Rahim *et al.*[9] Extracted from PET scans and not used in the *CT* feature set.

2.5.2 Morphological (shape) features

The morphological features (5) are defined as:

- **Volume**: Number of voxels in the tumour region multiplied by the dimension of voxels. Extracted from CT scans and used in all feature sets.

- **Size**: Maximum diameter of the tumour region. Extracted from CT scans, used in all feature sets.

- **Solidity**: Ratio of the number of voxels in the tumour region to the number of voxels in the 3D convex hull of the tumour region (smallest polyhedron containing the tumour region). Extracted from CT scans and used in all feature sets.

- **Eccentricity**: The ellipsoid that best fits the tumour region is first computed using the framework of Li & Griffiths[10]. The eccentricity is then given by $\left[1 - a \times b/c^2\right]^{1/2}$, where c is the longest semi-principal axes of the ellipsoid, and a and b are the second and third longest semi-principal axes of the ellipsoid. Extracted from CT scans and used in all feature sets.

- **Compactness**:

  $$compactness = \frac{V}{\sqrt{\pi} A^{3/2}}$$

  Where V denotes the volume and A the surface area of the ROI. Extracted from CT scans and used in all feature sets.

2.5.3 Texture features

In this work, a total of 40 different texture features were extracted from both the PET and CT volumes: 9 GLCM texture features, 13 GLRLM texture features, 13 GLSZM texture features and 5 NGTDM texture features. All 40 texture features from both PET and CT volumes were extracted using 40 different parameters. These 40 texture extraction parameters constitutes all possible combinations of the following different parameter values from 3 parameter types:

- **Isotropic voxel size** (5): This parameter is denoted as "*Scale*". Prior to the computation of texture features, all volumes were resampled to an isotropic voxel size set to a desired resolution using cubic interpolation and ROI masks using nearest neighbour interpolation. *Scale* values of 1 mm, 2 mm, 3 mm, 4 mm and 5 mm were tested in this work. For example, if the desired *Scale* was set to 5 mm, a PET volume with voxels of size $5.47 \times 5.47 \times 3.27$ mm^3 was isotropically resampled to voxels of size $5 \times 5 \times 5$ mm^3.

- **Quantization algorithm** (2): This parameter is denoted as "*Quant. algo.*" Prior to the computation of texture features, the full range of gray levels of the tumour region was quantized to a smaller number of gray levels N_g. *Equal-probability* and *Uniform* quantization algorithms were implemented in this work. *Equal-probability* quantization attempts to define decision thresholds in the volume such that the number of voxels is the same for all different gray levels. An in-house algorithm was implemented in MATLAB® using the function *histeq.m* to ensure a monotonic transformation of the intensity histograms. *Uniform* quantization is common practice in texture analysis: it uniformly divides the range of intensities in the volume into N_g gray-level bins.

- **Number of gray levels** (4): This parameter is denoted as "N_g". It represents the final number of gray levels in the quantized volume. N_g values of 8, 16, 32 and 64 were tested in this work.

More details about texture extraction parameters can also be found in the work of Vallières *et al.*[1].

Gray-Level Co-occurence Matrix (GLCM) features

Let P define the GLCM of a quantized volume $V(x, y, z)$ with isotropic voxel size. $P(i, j)$ represents the number of times voxels of gray-level i were neighbours with voxels of gray-level j in V, and N_g represents the pre-defined number of quantized gray-levels set in V. Only one GLCM of size $N_g \times N_g$ is computed per volume V by simultaneously adding up the frequency of co-occurences of all voxels with their 26-connected neighbours in 3D space, with all voxels (*including* the peripheral region) considered once as a center voxel (as defined by Haralick *et al.*[11], thus always using $d = 1$). To account for discretization length differences, neighbours at a distance of $\sqrt{3}$ voxels around a center voxel increment the

GLCM by a value of $\sqrt{3}$, neighbours at a distance of $\sqrt{2}$ voxels around a center voxel increment the GLCM by a value of $\sqrt{2}$, and neighbours at a distance of 1 voxel around a center voxel increment the GLCM by a value of 1 (as initially suggested by Vallières *et al.*[1]). The entry (i, j) of the of the normalized GLCM is then defined as:

$$p(i,j) = \frac{P(i,j)}{\sum_{i=1}^{N_g} \sum_{j=1}^{N_g} P(i,j)}.$$

The following quantities are also defined:

$$\mu_i = \sum_{i=1}^{N_g} i \sum_{j=1}^{N_g} p(i,j), \qquad \mu_j = \sum_{j=1}^{N_g} j \sum_{i=1}^{N_g} p(i,j),$$

$$\sigma_i = \sum_{i=1}^{N_g} (i-\mu_i)^2 \sum_{j=1}^{N_g} p(i,j), \qquad \sigma_j = \sum_{j=1}^{N_g} (j-\mu_j)^2 \sum_{i=1}^{N_g} p(i,j).$$

The GLCM texture features (9) are then defined as:

- **Energy**[11]:

$$energy = \sum_{i=1}^{N_g} \sum_{j=1}^{N_g} [p(i,j)]^2$$

- **Contrast**[11]:

$$contrast = \sum_{i=1}^{N_g} \sum_{j=1}^{N_g} (i-j)^2 \, p(i,j)$$

- **Correlation** (adapted from ref.[11]):

$$correlation = \sum_{i=1}^{N_g} \sum_{j=1}^{N_g} \frac{(i-\mu_i)\,(j-\mu_j)\,p(i,j)}{\sigma_i\,\sigma_j}$$

- **Homogeneity** (adapted from ref.[11]):

$$homogeneity = \sum_{i=1}^{N_g} \sum_{j=1}^{N_g} \frac{p(i,j)}{1+|i-j|}$$

- **Variance** (adapted from ref.[11]):

$$variance = \frac{1}{N_g \times N_g} \sum_{i=1}^{N_g} \sum_{j=1}^{N_g} \left[(i-\mu_i)^2\, p(i,j) + (j-\mu_j)^2\, p(i,j)\right]$$

- **Sum Average** (adapted from ref.[11]):

$$sum\ average = \frac{1}{N_g \times N_g} \sum_{i=1}^{N_g} \sum_{j=1}^{N_g} [i\, p(i,j) + j\, p(i,j)]$$

- **Entropy**[11]:

$$entropy = -\sum_{i=1}^{N_g} \sum_{j=1}^{N_g} p(i,j) \log_2\big(p(i,j)\big)$$

- **Dissimilarity**[12]:

$$dissimilarity = \sum_{i=1}^{N_g} \sum_{j=1}^{N_g} |i-j|\, p(i,j)$$

- **Autocorrelation**[13]:

$$autocorrelation = \sum_{i=1}^{N_g} \sum_{j=1}^{N_g} ij\, p(i,j)$$

Gray-Level Run-Length Matrix (GLRLM) features
Let P define the GLRLM of a quantized volume $V(x,y,z)$ with isotropic voxel size. $P(i,j)$ represents the number of runs of gray-level i and of length j in V, N_g represents the pre-defined number of quantized gray-levels set in V, and L_r represents the length of the longest run (of any gray-level) in V. Only one GLRLM of size $N_g \times L_r$ is computed per volume V by simultaneously adding up all possible longest run-lengths in the 13 directions of 3D space (one voxel can be part of multiple runs in different directions, but can be part of only one run in a given direction). A MATLAB® toolbox created by Xunkai Wei[14] computes GLRLMs from 2D images, and it can be used to facilitate the computation of GLRLMs from 3D volumes. To account for discretization length differences, runs constructed from voxels separated by a distance of $\sqrt{3}$ increment the GLRLM by a value of $\sqrt{3}$, runs constructed from voxels separated by a distance of $\sqrt{2}$ increment the GLRLM by a value of $\sqrt{2}$, and runs constructed from voxels separated by a distance of 1 increment the GLRLM by a value of 1 (as initially suggested by Vallières *et al.*[1]). The entry (i,j) of the of the normalized GLRLM is then defined as:

$$p(i,j) = \frac{P(i,j)}{\sum_{i=1}^{N_g} \sum_{j=1}^{L_r} P(i,j)}.$$

The following quantities are also defined:

$$\mu_i = \sum_{i=1}^{N_g} i \sum_{j=1}^{L_r} p(i,j), \qquad \mu_j = \sum_{j=1}^{L_r} j \sum_{i=1}^{N_g} p(i,j).$$

The GLRLM texture features (13) are then defined as:

- **Short Run Emphasis (SRE)**[15]:

$$SRE = \sum_{i=1}^{N_g} \sum_{j=1}^{L_r} \frac{p(i,j)}{j^2}$$

- **Long Run Emphasis (LRE)**[15]:

$$LRE = \sum_{i=1}^{N_g} \sum_{j=1}^{L_r} j^2 \, p(i,j)$$

- **Gray-Level Nonuniformity (GLN)** (adapted from ref.[15]):

$$GLN = \sum_{i=1}^{N_g} \left(\sum_{j=1}^{L_r} p(i,j) \right)^2$$

- **Run-Length Nonuniformity (RLN)** (adapted from ref.[15]):

$$RLN = \sum_{j=1}^{L_r} \left(\sum_{i=1}^{N_g} p(i,j) \right)^2$$

- **Run Percentage (RP)** (adapted from ref.[15]):

$$RP = \frac{\sum_{i=1}^{N_g} \sum_{j=1}^{L_r} p(i,j)}{\sum_{j=1}^{L_r} j \sum_{i=1}^{N_g} p(i,j)}$$

- **Low Gray-Level Run Emphasis (LGRE)**[16]:

$$LGRE = \sum_{i=1}^{N_g} \sum_{j=1}^{L_r} \frac{p(i,j)}{i^2}$$

- **High Gray-Level Run Emphasis (HGRE)**[16]:

$$HGRE = \sum_{i=1}^{N_g} \sum_{j=1}^{L_r} i^2 \, p(i,j)$$

- **Short Run Low Gray-Level Emphasis (SRLGE)**[17]:

$$SRLGE = \sum_{i=1}^{N_g} \sum_{j=1}^{L_r} \frac{p(i,j)}{i^2 j^2}$$

- **Short Run High Gray-Level Emphasis (SRHGE)**[17]:

$$SRHGE = \sum_{i=1}^{N_g} \sum_{j=1}^{L_r} \frac{i^2\, p(i,j)}{j^2}$$

- **Long Run Low Gray-Level Emphasis (LRLGE)**[17]:

$$LRLGE = \sum_{i=1}^{N_g} \sum_{j=1}^{L_r} \frac{j^2\, p(i,j)}{i^2}$$

- **Long Run High Gray-Level Emphasis (LRHGE)**[17]:

$$LRHGE = \sum_{i=1}^{N_g} \sum_{j=1}^{L_r} i^2 j^2\, p(i,j)$$

- **Gray-Level Variance (GLV)** (adapted from ref.[18]):

$$GLV = \frac{1}{N_g \times L_r} \sum_{i=1}^{N_g} \sum_{j=1}^{L_r} \left(i\, p(i,j) - \mu_i\right)^2$$

- **Run-Length Variance (RLV)** (adapted from ref.[18]):

$$RLV = \frac{1}{N_g \times L_r} \sum_{i=1}^{N_g} \sum_{j=1}^{L_r} \left(j\, p(i,j) - \mu_j\right)^2$$

Gray-Level Size Zone Matrix (GLSZM) features

Let P define the GLSZM of a quantized volume $V(x, y, z)$ with isotropic voxel size. $P(i, j)$ represents the number of 3D zones of gray-levels i and of size j in V, N_g represents the pre-defined number of quantized gray-levels set in V, and L_z represents the size of the largest zone (of any gray-level) in V. One GLSZM of size $N_g \times L_z$ is computed per volume V by adding up all possible largest zone-sizes, with zones constructed from 26-connected neighbours of the same gray-level in 3D space (one voxel can be part of only one zone). The entry (i, j) of the normalized GLSZM is then defined as:

$$p(i,j) = \frac{P(i,j)}{\sum_{i=1}^{N_g} \sum_{j=1}^{L_z} P(i,j)}.$$

The following quantities are also defined:

$$\mu_i = \sum_{i=1}^{N_g} i \sum_{j=1}^{L_z} p(i,j), \qquad \mu_j = \sum_{j=1}^{L_z} j \sum_{i=1}^{N_g} p(i,j).$$

The GLSZM texture features (13) are then defined as:

- **Small Zone Emphasis (SZE)**[15,18]:

$$SZE = \sum_{i=1}^{N_g} \sum_{j=1}^{L_z} \frac{p(i,j)}{j^2}$$

- **Large Zone Emphasis (LZE)**[15,18]:

$$LZE = \sum_{i=1}^{N_g} \sum_{j=1}^{L_z} j^2 \, p(i,j)$$

- **Gray-Level Nonuniformity (GLN)** (adapted from refs.[15,18]):

$$GLN = \sum_{i=1}^{N_g} \left(\sum_{j=1}^{L_z} p(i,j) \right)^2$$

- **Zone-Size Nonuniformity (ZSN)** (adapted from refs.[15,18]):

$$ZSN = \sum_{j=1}^{L_z} \left(\sum_{i=1}^{N_g} p(i,j) \right)^2$$

- **Zone Percentage (RP)** (adapted from refs.[15,18]):

$$ZP = \frac{\sum_{i=1}^{N_g} \sum_{j=1}^{L_z} p(i,j)}{\sum_{j=1}^{L_z} j \sum_{i=1}^{N_g} p(i,j)}$$

- **Low Gray-Level Zone Emphasis (LGZE)**[16,18]:

$$LGZE = \sum_{i=1}^{N_g} \sum_{j=1}^{L_z} \frac{p(i,j)}{i^2}$$

- **High Gray-Level Zone Emphasis (HGZE)**[16,18]:

$$HGZE = \sum_{i=1}^{N_g} \sum_{j=1}^{L_z} i^2 \, p(i,j)$$

- **Small Zone Low Gray-Level Emphasis (SZLGE)**[17,18]:

$$SZLGE = \sum_{i=1}^{N_g} \sum_{j=1}^{L_z} \frac{p(i,j)}{i^2 j^2}$$

- **Small Zone High Gray-Level Emphasis (SZHGE)**[17,18]:

$$SZHGE = \sum_{i=1}^{N_g} \sum_{j=1}^{L_z} \frac{i^2\, p(i,j)}{j^2}$$

- **Large Zone Low Gray-Level Emphasis (LZLGE)**[17,18]:

$$LZLGE = \sum_{i=1}^{N_g} \sum_{j=1}^{L_z} \frac{j^2\, p(i,j)}{i^2}$$

- **Large Zone High Gray-Level Emphasis (LZHGE)**[17,18]:

$$LZHGE = \sum_{i=1}^{N_g} \sum_{j=1}^{L_z} i^2 j^2\, p(i,j)$$

- **Gray-Level Variance (GLV)** (adapted from ref.[18]):

$$GLV = \frac{1}{N_g \times L_z} \sum_{i=1}^{N_g} \sum_{j=1}^{L_z} \left(i\, p(i,j) - \mu_i\right)^2$$

- **Zone-Size Variance (ZSV)** (adapted from ref.[18]):

$$ZSV = \frac{1}{N_g \times L_z} \sum_{i=1}^{N_g} \sum_{j=1}^{L_z} \left(j\, p(i,j) - \mu_j\right)^2$$

Neighbourhood Gray-Tone Difference Matrix (NGTDM) features
Let $P(i)$ define the NGTDM of a quantized volume $V(x,y,z)$ with isotropic voxel size. $P(i)$ represents the summation of the gray-level differences between all voxels with gray-level i and the average gray-level of their 26-connected neighbours in 3D space. N_g represents the pre-defined number of quantized gray-levels set in V, and $(N_g)_{eff}$ is the effective number of gray-levels in V, with $(N_g)_{eff} < N_g$ (let the vector of gray-levels values in V be denoted as $\mathbf{g} = g(1), g(2), \ldots, g(N_g)$; some gray-levels excluding $g(1)$ and $g(N_g)$ may not appear in V due to different quantization schemes). One NGTDM of size $N_g \times 1$ is computed per volume V. To account for discretization length differences, all averages around a center voxel located at position (j,k,l) in V are performed such that the neighbours at a distance of $\sqrt{3}$ voxels are given a weight of $1/\sqrt{3}$, the neighbours at a distance of $\sqrt{2}$ voxels are given a weight of $1/\sqrt{2}$, and the neighbours at a distance of 1 voxel are given a weight of 1 (as initially suggested by Vallières *et al.*[1]). The ith entry of the NGTDM is then defined as:

$$P(i) = \begin{cases} \sum_{\text{all voxels} \in \{N_i\}} |i - \overline{A}_i| & \text{if } N_i > 0, \\ 0 & \text{if } N_i = 0. \end{cases}$$

where $\{N_i\}$ is the set of all voxels with gray-level i in V (*including* the peripheral region), N_i is the number of voxels with gray-level i in V, and $\overline{A}_i$ is the average gray-level of the 26-connected neighbours around a center voxel with gray-level i and located at position (j,k,l) in V such that:

$$\overline{A}_i = \overline{A}(j,k,l) = \frac{\sum_{m=-1}^{m=1}\sum_{n=-1}^{n=1}\sum_{o=-1}^{o=1} w_{m,n,o}\cdot V(j+m,k+n,l+o)}{\sum_{m=-1}^{m=1}\sum_{n=-1}^{n=1}\sum_{o=-1}^{o=1} w_{m,n,o}},$$

$$\text{where}\quad w_{m,n,o} = \begin{cases} 1 & \text{if } |j-m|+|k-n|+|l-o| = 1, \\ \frac{1}{\sqrt{2}} & \text{if } |j-m|+|k-n|+|l-o| = 2, \\ \frac{1}{\sqrt{3}} & \text{if } |j-m|+|k-n|+|l-o| = 3, \\ 0 & \text{if } V(j+m,k+n,l+o) \text{ is undefined.} \end{cases}$$

The following quantity is also defined:

$$n_i = \frac{N_i}{N}.$$

where N is the total number of voxels in V.

The NGTDM texture features (5) are then defined as:

- **Coarseness**[19]:

$$coarseness = \left[\epsilon + \sum_{i=1}^{N_g} n_i\, P(i)\right]^{-1}$$

  where ϵ is a small number to prevent *coarseness* becoming infinite.

- **Contrast**[19]:

$$contrast = \left[\frac{1}{(N_g)_{eff}\,\left[(N_g)_{eff}-1\right]}\sum_{i=1}^{N_g}\sum_{j=1}^{N_g} n_i\, n_j\,(i-j)^2\right]\left[\frac{1}{N}\sum_{i=1}^{N_g} P(i)\right]$$

- **Busyness**[19]:

$$busyness = \frac{\sum_{i=1}^{N_g} n_i\, P(i)}{\sum_{i=1}^{N_g}\sum_{j=1}^{N_g}(i\, n_i - j\, n_j)}, \quad n_i \neq 0, n_j \neq 0$$

- **Complexity**[19]:

$$complexity = \sum_{i=1}^{N_g}\sum_{j=1}^{N_g} \frac{|i-j|\left[n_i\, P(i) + n_j\, P(j)\right]}{N\,(n_i+n_j)}, \quad n_i \neq 0, n_j \neq 0$$

- **Strength**[19]:

$$strength = \frac{\sum_{i=1}^{N_g} \sum_{j=1}^{N_g} (n_i + n_j)\,(i-j)^2}{\left[\epsilon + \sum_{i=1}^{N_g} P(i)\right]}, \quad n_i \neq 0, n_j \neq 0$$

where ϵ is a small number to prevent *strength* becoming infinite.

2.5.4 Online resources

MATLAB® software code for computing radiomic features is freely shared under the GNU General Public License at: https://github.com/mvallieres/radiomics.

2.6 Computation of the radiomic signature

2.6.1 Original radiomic signature

This section details how the original radiomic signature proposed by Aerts & Velazquez *et al.*[13] was computed from CT scans in the current work. In their original work, Aerts & Velazquez *et al.*[13] extracted the four features of the radiomic signature on CT images with voxels of size $1 \times 1 \times 3$ mm^3. In the current work, the CT images were thus first resampled to the same voxel size of $1 \times 1 \times 3$ mm^3 using cubic interpolation. The four features of the radiomic signature were then computed from the region of interest of the tumour as defined by the "$\text{GTV}_{\text{primary}} + \text{GTV}_{\text{lymph nodes}}$" contours (ROI) as follows:

1. **Energy**
   Let $\mathbf{X}$ define the vector of Hounsfield Units (HUs) from CT scans for the N voxels of the ROI. The feature *energy* is then defined as:

$$energy = \sum_{i=1}^{N} X(i)^2$$

2. **Compactness**
   Let V be the volume in mm^3 and A be the surface area in mm^2 of the ROI. The feature *compactness* is then defined as:

$$compactness = \frac{V}{\sqrt{\pi} A^{2/3}}$$

3. **GLN**
   To compute the Gray-Level Nonuniformity (GLN) texture feature similarly to the work of Aerts & Velazquez *et al.*[13], the ROI was first quantized to a number of gray levels N_g^p different for each patient p. For CT scans, bins of 25 HUs were created using a lower limit of 0 HU to the intensity range of the bins such that all voxels within the ROI with $-1000 \leq \text{HU} < 25$ were assigned to gray-level 1, all voxels within the ROI with $25 \leq \text{HU} < 50$ were assigned to gray-level 2, etc.

   Then, let $P_\delta(i,j)$ define the directional GLRLM of the quantized ROI, where δ denotes one of the 13 directions around a center voxel in 3D space. Similarly to what is described in the previous section, $P_\delta(i,j)$ represents the number of runs of gray-level i and of length j, and L_r represents the length of the longest run (of any gray-level) in the quantized ROI **for direction** δ. The GLN_δ for direction δ is then defined as:

$$GLN_\delta = \frac{\sum_{i=1}^{N_g^p} \left(\sum_{j=1}^{L_r} P_\delta(i,j) \right)^2}{\sum_{i=1}^{N_g^p} \sum_{j=1}^{L_r} P_\delta(i,j)}$$

Finally, the GLN texture feature is calculated as:

$$GLN = \frac{1}{13}\sum_{\delta=1}^{13} GLN_{\delta}$$

4. **GLN_HLH**
This texture feature is obtained by computing the GLN texture feature described above (feature 3) in the HLH sub-band of the first decomposition level of the 3D undecimated discrete wavelet transform performed using the wavelet basis function "Coiflet 1".

The HLH wavelet decomposition is traditionally obtained by applying a high-pass filter in the x-direction, a low-pass filter in the y-direction and a high-pass filter in the z-direction. For medical images, standard practice is to consider the reference coordinate system (RCS) of the DICOM protocol in order to unambiguously define the filter directions. Hence, for an axial CT volume of a patient in the DICOM RCS, the HLH wavelet decomposition would be obtained by applying a high-pass filter in the lef-right direction, a low-pass filter in the anterior-posterior direction and a high-pass filter in the inferior-superior direction.

However, in their original work, Aerts & Velazquez *et al.*[13] considered the MATLAB® conventions to define the directions of the filters. As a result, the HLH wavelet decomposition was obtained by applying a high-pass filter in the anterior-posterior direction, a low-pass filter in the left-right direction and a high-pass filter in the inferior-superior direction of axial CT images. The same filter directions as defined by MATLAB® conventions were thus also used for CT images in the current work.

Practically speaking, in this work, the undecimated wavelet transform was applied on the original ROI using the function *swt2* and the wavelet basis function "Coiflet 1" of MATLAB®. To achieve a 3D decomposition, the 2D undecimated discrete wavelet transform obtained with the *swt2* function was successively applied for all image planes of the ROI in the x-, y- and z-directions of the RCS, and the corresponding wavelet coefficients of all image planes were averaged. The resulting wavelet coefficients of the ROI corresponding to the HLH sub-band were then uniformly quantized to the same number of gray levels N_g^p (for a given patient p) as obtained with the computation of the standard GLN texture feature described above (feature 3). Finally, the GLN_HLH texture feature was obtained by computing the same GLN texture feature described above (feature 3) to the quantized ROI of the HLH wavelet sub-band.

COMPLETE MODEL. In one instance in our work, we directly tested in the testing set (H&N3 and H&N4; $n = 106$) a Cox regression model constructed using the original coefficients and median hazard ratio trained in the Lung1

cohort of the original work of Aerts & Velazquez *et al.*[13]. This complete Cox regression model $\lambda(\mathbf{x}_i)$ was applied as follows in our work:

$$\begin{aligned}\lambda(\mathbf{x}_i) = &\\ & -2.42e\text{-}11 \times \text{CT-Energy}\\ & +5.38e\text{-}03 \times \text{CT-Compactness}\\ & +1.47e\text{-}04 \times \text{CT-GLN}_{GLRLM}\\ & -9.39e\text{-}06 \times \text{CT-GLN\_HLH}_{GLRLM}\end{aligned}$$

with a median hazard ratio of 0.1191567. The greater $\lambda(\mathbf{x}_i)$, the worst the chanches of survival are.

2.6.2 Revised version of the radiomic signature

This section details three modifications applied to the original radiomic signature in order to obtain a revised version (other than the following modifications, the computation remained the same as described in the previous section):

1. **CT resampling**
   In order to obtain isotropic voxel size, the CT images were resampled to a voxel size of $1 \times 1 \times 1$ mm^3 using cubic interpolation.

2. **Compactness**
   The definition of *compactness* in the original radiomic signature uses $A^{2/3}$ in the denominator. This is most likely an error in the original paper of Aerts & Velazquez *et al.*[13], as $A^{3/2}$ is required to create a dimensionless feature. The feature *compactness* is thus hereby defined as:

$$compactness = \frac{V}{\sqrt{\pi}A^{3/2}}$$

3. **Computation of GLN and GLN_HLH**

   - Only one GLRLM is computed per CT volume by simultaneously adding up the 13 GLRLMs of all 3D directions. The GLRLM averaging technique used for the original radiomic signature basically results in an average of limited run-length measurements.
   - A normalized version of the GLN feature is used in this work. This feature is defined in section 2.5.3 of this document under the "GLRLM" heading. The original GLN feature as defined by Galloway[15] is not properly normalized and is thus dependent on the total number of runs in a given volume.

References

1. Vallières, M., Freeman, C. R., Skamene, S. R. & El Naqa, I. A radiomics model from joint FDG-PET and MRI texture features for the prediction of lung metastases in soft-tissue sarcomas of the extremities. *Phys. Med. Biol.* **60,** 5471–5496 (2015).
2. Efron, B. & Tibshirani, R. Improvements on cross-validation: the 632+ bootstrap method. *Journal of the American Statistical Association* **92,** 548–560 (June 1, 1997).
3. Sahiner, B., Chan, H.-P. & Hadjiiski, L. Classifier performance prediction for computer-aided diagnosis using a limited dataset. *Med. Phys.* **35,** 1559–1570 (Apr. 1, 2008).
4. El Naqa, I. *et al.* Dose response explorer: an integrated open-source tool for exploring and modelling radiotherapy dose-volume outcome relationships. *Phys. Med. Biol.* **51,** 5719–5735 (2006).
5. Schiller, T. W., Chen, Y., El Naqa, I. & Deasy, J. O. Modeling radiation-induced lung injury risk with an ensemble of support vector machines. *Neurocomputing* **73,** 1861–1867 (2010).
6. Reshef, D. N. *et al.* Detecting novel associations in large data sets. *Science* **334,** 1518–1524 (Dec. 16, 2011).
7. Bonner, J. A. *et al.* Radiotherapy plus cetuximab for squamous-cell carcinoma of the head and neck. *N. Engl. J. Med.* **354,** 567–578 (2006).
8. Van Velden, F. H. P. *et al.* Evaluation of a cumulative SUV-volume histogram method for parameterizing heterogeneous intratumoural FDG uptake in non-small cell lung cancer PET studies. *Eur. J. Nucl. Med. Mol. Imaging* **38,** 1636–1647 (2011).
9. Rahmim, A. *et al.* A novel metric for quantification of homogeneous and heterogeneous tumors in PET for enhanced clinical outcome prediction. *Phys. Med. Biol.* **61,** 227 (2016).
10. Li, Q. & Griffiths, J. G. *Least squares ellipsoid specific fitting* in *Proceedings of the Geometric Modeling and Processing 2004.* International Conference on Geometric Modeling and Processing (GMP 04) (Beijing, China, 2004), 335–340.
11. Haralick, R. M., Shanmugam, K. & Dinstein, I. Textural features for image classification. *IEEE Transactions on Systems, Man, and Cybernetics* **SMC-3,** 610–621 (1973).
12. Thibault, G. *Indices de formes et de textures: de la 2D vers la 3D.* PhD thesis (Université AIX-Marseille, Marseille, France, 2009).
13. Aerts, H. J. W. L. *et al.* Decoding tumour phenotype by noninvasive imaging using a quantitative radiomics approach. *Nat. Commun.* **5,** 4006 (2014).
14. Wei, X. *Gray Level Run Length Matrix Toolbox* version 1.0. 2007.

15. Galloway, M. M. Texture analysis using gray level run lengths. *Computer Graphics and Image Processing* **4,** 172–179 (1975).

16. Chu, A., Sehgal, C. M. & Greenleaf, J. F. Use of gray value distribution of run lengths for texture analysis. *Pattern Recognition Letters* **11,** 415–419 (1990).

17. Dasarathy, B. V. & Holder, E. B. Image characterizations based on joint gray level–run length distributions. *Pattern Recognition Letters* **12,** 497–502 (1991).

18. Thibault, G. *et al. Texture indexes and gray level size zone matrix: application to cell nuclei classification* in *Proceedings of the Pattern Recognition and Information Processing 2009.* International Conference on Pattern Recognition and Information Processing (PRIP '09) (Minsk, Belarus, 2009), 140–145.

19. Amadasun, M. & King, R. Textural features corresponding to textural properties. *IEEE Transactions on Systems, Man, and Cybernetics* **19,** 1264–1274 (1989).
